# Putting ChatGPT’s Medical Advice to the (Turing) Test: Survey Study

**DOI:** 10.2196/46939

**Published:** 2023-07-10

**Authors:** Oded Nov, Nina Singh, Devin Mann

**Affiliations:** 1 Department of Technology Management Tandon School of Engineering New York University New York, NY United States; 2 Department of Population Health Grossman School of Medicine New York University New York, NY United States; 3 Medical Center Information Technology Langone Health New York University New York, NY United States

**Keywords:** artificial intelligence, AI, ChatGPT, Chat Generative Pre-trained Transformer, large language model, patient-provider interaction, chatbot, feasibility, ethics, privacy, language model, machine learning

## Abstract

**Background:**

Chatbots are being piloted to draft responses to patient questions, but patients’ ability to distinguish between provider and chatbot responses and patients’ trust in chatbots’ functions are not well established.

**Objective:**

This study aimed to assess the feasibility of using ChatGPT (Chat Generative Pre-trained Transformer) or a similar artificial intelligence–based chatbot for patient-provider communication.

**Methods:**

A survey study was conducted in January 2023. Ten representative, nonadministrative patient-provider interactions were extracted from the electronic health record. Patients’ questions were entered into ChatGPT with a request for the chatbot to respond using approximately the same word count as the human provider’s response. In the survey, each patient question was followed by a provider- or ChatGPT-generated response. Participants were informed that 5 responses were provider generated and 5 were chatbot generated. Participants were asked—and incentivized financially—to correctly identify the response source. Participants were also asked about their trust in chatbots’ functions in patient-provider communication, using a Likert scale from 1-5.

**Results:**

A US-representative sample of 430 study participants aged 18 and older were recruited on Prolific, a crowdsourcing platform for academic studies. In all, 426 participants filled out the full survey. After removing participants who spent less than 3 minutes on the survey, 392 respondents remained. Overall, 53.3% (209/392) of respondents analyzed were women, and the average age was 47.1 (range 18-91) years. The correct classification of responses ranged between 49% (192/392) to 85.7% (336/392) for different questions. On average, chatbot responses were identified correctly in 65.5% (1284/1960) of the cases, and human provider responses were identified correctly in 65.1% (1276/1960) of the cases. On average, responses toward patients’ trust in chatbots’ functions were weakly positive (mean Likert score 3.4 out of 5), with lower trust as the health-related complexity of the task in the questions increased.

**Conclusions:**

ChatGPT responses to patient questions were weakly distinguishable from provider responses. Laypeople appear to trust the use of chatbots to answer lower-risk health questions. It is important to continue studying patient-chatbot interaction as chatbots move from administrative to more clinical roles in health care.

## Introduction

Advances in large language models (LLMs) have enabled dramatic improvements in the quality of artificial intelligence (AI)–generated conversations. Recently, the launch of ChatGPT (Chat Generative Pre-trained Transformer; OpenAI) [1] has prompted a surge of interest in AI-based chatbots, both from the health care field [2,3] and the general public [4,5]. Several health care systems, including University of California San Diego Health and University of Wisconsin Health, have already announced pilots of using the underlying Generative Pre-trained Transformer (GPT) technology as a means of drafting initial responses to patient portal messages [6]. Other health care systems, including Stanford Health Care, are also preparing for pilots of GPT-drafted patient portal message responses [6].

This study assessed the feasibility of using ChatGPT or similar AI-based chatbots for answering patient portal messages directed at health care providers. ChatGPT is a chatbot created by OpenAI that is based on the LLM known as GPT [1]. At a high level, it was trained to predict the most probable next word using a large body of text data from the internet, and it was optimized to respond to user queries using reinforcement learning with human feedback on its responses to questions. Although it is generally able to generate humanlike and accurate text, LLMs such as ChatGPT have several limitations. These include biases from the underlying data (eg, social biases such as racism and sexism) [7,8], the ability to “hallucinate” information that is untrue [9], and the lack of mental models that would allow for true reasoning rather than simply probabilistic text generation (leading it to make errors in response to queries such as simple arithmetic problems) [10].

Using ChatGPT or similar AI-based chatbots to respond to patient portal messages is of interest given the recently launched pilots, the increasing burden of patient messages being delivered to providers [11], and the association between increased electronic health record (EHR) work and provider burnout [12,13]. Moreover, providers are generally not allocated time or reimbursement for answering patient messages. In an age when patients increasingly expect providers to be digitally accessible, it is likely that patient message load will continue to increase. As the technology behind AI-based chatbots matures, the time is ripe for exploring chatbots’ potential role in patient-provider communication.

Recent studies have had health care professionals judge ChatGPT’s responses to health-related questions [14-16], with findings such as 84% of answers to cardiovascular disease prevention questions being appropriate [15] and ChatGPT overall scoring higher for quality and empathy than health care providers [16]. Fewer studies have examined patient attitudes toward ChatGPT providing responses to health-related questions [17]. Here, we sought to understand how patients may perceive AI chatbot–generated responses to their questions. We reported on the ability of members of the public to distinguish between AI- and provider-generated responses to patients’ health questions. Further, we characterized participants’ trust in chatbots’ functions. Finally, we discussed the possible implications of the adoption of AI-based chatbots in patient messaging portals.

Notably, we were not trying to distinguish whether AI- or human-generated responses are a better solution for patients. Rather, we studied whether patients can tell that the response is coming from AI versus a provider and whether they trust AI, which are separate questions.

## Methods

### Overview

Ten representative, nonadministrative patient-provider interactions from one of the authors were extracted from the patient-provider interaction module of the EHR. All identifying details were removed, and typos in the provider’s response were fixed. Patients’ questions were entered into ChatGPT on January 19, 2023, with a request to respond using approximately the same word count as the provider’s response (see Textbox 1). Chatbot response text that recommended consultation with the patient’s health care provider was removed. The response accuracy of the human and ChatGPT responses were not evaluated to provide as close as possible to an in-the-wild experience for participants.

The 10 questions and responses were presented to a US-representative sample of 430 people aged 18 years and older who were recruited on Prolific, a crowdsourcing platform for academic studies. Participants provided written informed consent to take part in the study.

Participants were informed that 5 of the responses were written by a human provider and 5 were generated by an AI-based chatbot. For each participant, each patient question was followed by either a provider- or ChatGPT-generated response. Participants were asked to determine which responses were written by a provider and which were generated by a chatbot. The setup of 5 human responses versus 5 chatbot responses follows Fisher’s [18] seminal work on experimental design, which recommends an equal distribution of items and that participants be told in advance of the distribution. In doing so, we (1) establish a uniform prior belief in the probability associated with each advice source, (2) promote independent decision-making by participants regarding individual responses without considering other questions, and (3) avoid any influence that could sway participants’ preferences toward a specific advice source. The order of the 10 questions and answers, as well as the order of the choices presented to participants, were randomized. Participants were incentivized financially to distinguish between human and chatbot responses (US $2 baseline compensation, with up to a US $3 bonus for answering questions correctly).

Participants were then asked questions about their trust in chatbots’ use in patient-provider communication using a Likert scale from 1-5 (see the *Results* section). They were asked about their trust in chatbots to provide different types of services (logistical information, preventative care advice, diagnostic advice, and treatment advice), their trust in AI chatbots to answer health questions compared to a Google search, and their overall trust in AI chatbots to help them make better health decisions.

With respect to distinguishability, a chi-squared test for proportions was used to determine if there was a difference in the proportion of correct identification by men versus women. A chi-square test for goodness of fit was used to investigate whether there were variations in the proportion of correct identification across different participant age groups. Similarly, differences across age and gender in participants’ response to the survey’s trust questions were analyzed using ANOVA. Across all tests, results were considered significant if *P*<.05.

**Sample patient question (A) and answers (B and C).**

**A. Patient question**

You indicated that based on lab tests conducted last month, my blood sugar levels were at a pre-diabetic stage. Could you provide me with steps I can take to reduce those levels, including any specific dietary recommendations. Thanks very much. 

**B. Human response**

It comes down to weight loss with some physical activity which can help as well. How you achieve weight loss really depends on each person. Rules of thumb are to reduce carbohydrate intake both from simple sugars as well as starches like bread pasta and rice. In addition, limiting any juices or sweets helps as well. But most importantly is really increasing the amount of healthy fruit and vegetables and other high nutrient density foods and just limiting the portion sizes of the foods as well. Many people appreciate a more structured program for reducing the risk of diabetes and if you look at the federal government’s National diabetes prevention program they have lots of online materials and there are often group classes at YMCA’s or other locations in the community.

**C. Chatbot response**

To reduce your pre-diabetic blood sugar levels, the first step is to focus on making lifestyle changes. Specifically, you should focus on your diet and exercise. Eating a diet high in whole, unprocessed foods such as fruits, vegetables and whole grains, while limiting your intake of added sugars, saturated fats, and processed foods will help. Incorporating more protein-rich foods such as lean meats, fish, and beans and eating small, frequent meals throughout the day rather than a few large ones can also help. In terms of exercise, try to aim for at least 30 minutes of moderate physical activity per day, such as brisk walking, cycling or swimming. It is also very important to maintain a healthy weight, as being overweight or obese can increase your risk of developing type 2 diabetes. I recommend speaking with a registered dietitian or nutritionist who can help you create a personalized eating plan.

### Ethical Considerations

This study was certified and filed as a Quality Improvement study per NYU Langone Health’s Quality Improvement self-certification protocol. As a Quality Improvement study, institutional review board approval is not needed.

## Results

Overall, 426 participants filled out the full survey. After removing participants who spent less than 3 minutes on the survey, 392 survey responses were used in the analysis. Of the 392 respondents, 53.3% (n=209) were women, and the average age was 47.1 (SD 16.0) years.

The responses to patient questions varied widely in the participants’ ability to identify whether they were written by a human or chatbot, ranging between 49% (192/392) to 85.7% (336/392) for different questions. Each participant received a score between 0-10 based on the number of responses they identified correctly (Multimedia Appendix 1). On average, chatbot responses were identified correctly in 65.5% (1284/1960) of the cases, and human provider responses were identified correctly in 65.1% (1276/1960) of the cases. No substantial differences were found in response distinguishability or trust by demographic characteristics.

On average, patients trusted chatbots (Table 1), yet trust was lower as the health-related complexity of the task in the questions increased. Logistical questions (eg, scheduling appointments and insurance questions) had the highest trust rating (mean Likert score 3.94, SD 0.92), followed by preventative care (eg, vaccines and cancer screenings; mean Likert score 3.52, SD 1.10). Diagnostic and treatment advice had the lowest trust ratings (mean Likert scores 2.90, SD 1.14 and 2.89, SD 1.12, respectively). No significant correlations were found between trust in health chatbots and demographics or the ability to correctly identify chatbot versus human responses (all *P*>.05).

**Table 1:** Health chatbot trust questions and responses.

Question	Patients with Likert response ≥4 (n=392), n (%)	Likert response (range 1-5), mean (SD)
I could trust answers from a health chatbot about logistical questions (such as scheduling appointments, insurance questions, medication requests).	312 (79.6)	3.94 (0.92)
I could trust a chatbot to provide advice about preventative care, such as vaccines, or cancer screenings.	248 (63.3)	3.52 (1.10)
I could trust a chatbot to provide diagnostic advice about symptoms.	152 (38.8)	2.90 (1.14)
I could trust a chatbot to provide treatment advice.	150 (38.3)	2.89 (1.12)
AI^a^ chatbots can be a more trustworthy alternative to Google to answer my health questions.	232 (59.2)	3.56 (1.02)
Health chatbots could help me make better decisions.	236 (60.2)	3.49 (0.91)

^a^AI: artificial intelligence.

## Discussion

### Principal Findings

Patients increasingly expect *consumer-grade* health care experiences that mirror their experiences with the rest of their digital life. They want omnichannel and interactive communication, frictionless access to care, and personalized education. The resulting overwhelming volume of patient portal messages highlights an opportunity for chatbots to assist health care providers, one that is already being acted upon by several large health care systems [6]. Early research on provider perception of these chatbot-generated responses has revealed high degrees of appropriateness [15] and has even revealed higher quality and empathy ratings than human-generated responses [16]. However, whether patients view chatbot communication as comparable to communication with human providers requires empirical investigation [19-21].

In this study of a US-representative sample, compared to the benchmark of 50% representing random distinguishability and 100% representing perfect distinguishability, laypeople found responses from an AI-based chatbot to be weakly distinguishable from those from a human provider. Notably, there was very little difference between the distinguishability rate of human versus chatbot responses (65.5 vs 65.1%).

It is likely that in the near future, the level of indistinguishability we found will represent a lower bound of performance, as chatbots trained on medical data specifically, or prompted with medical queries, will likely be less distinguishable [14]. Another possible future development is for chatbots to reach a superhuman level as seen in other medical domains [22]. The emerging group of vendors designing optimized prompt libraries for health systems is likely to further improve chatbots’ performance on health-related questions (eg, DocsGPT [23]). It is important to note that products based on LLMs, such as ChatGPT, merely provide text that resembles good medical advice, and it is only with the addition of medical knowledge that useful health care provider–level advice could be provided.

Respondents’ trust in chatbots’ functions were mildly positive. Notably, there was a lower level of trust in chatbots as the medical complexity of the task increased, with the highest acceptance for administrative tasks such as scheduling appointments and the lowest acceptance for treatment advice. This is broadly consistent with prior studies [17,24]. In particular, a recent study of user intentions to use ChatGPT for self-diagnosis found that higher performance expectancy and positive risk-reward appraisals were associated with improved perception of decision-making outcomes [17]. This improved perception in turn positively impacted participant intentions to use ChatGPT for self-diagnosis (78% of the 476 participants indicated that they were willing to do so) [17].

Our study suggests that participants are overall willing to receive health advice from a chatbot (especially for low-risk topics) and are only weakly able to distinguish between ChatGPT- versus human-generated responses. Based on our findings, identifying appropriate scenarios for deploying chatbots within health care systems is an important next step. Although chatbots are widely used in health care administrative tasks (eg, scheduling), optimal clinical use cases are still emerging [25]. Chatbots have been developed and deployed for highly specialized clinical scenarios such as symptom triage and postchemotherapy education [26]. More generalized chatbots that are similar to ChatGPT represent a new opportunity to use chatbots in support of more common chronic disease management for conditions such as hypertension, diabetes, and asthma. Health care providers’ work may be transformed by using the products of generative AI (such as chatbots’ output) as raw material to construct patient-provider interaction, including advice, the explanation of test results, the discussion of side effects, and many other types of interactions that currently require a human health care provider. For example, chatbots could be deployed with home blood pressure monitoring to support patient questions about treatment plans, medication titrations, and potential side effects [27].

Potential deployment models include chatbots that directly interact with patients (eg, through patient portals) or serve as clinician assistants, generating draft text or transforming clinician documentation into more patient-friendly versions. For health care providers’ work, this would lead to a shift in focus from the *creation* of health care advice to the *curation* of advice in response to patient messages. Of note, it is critical that providers stay alert when curating rather than simply accepting the models’ answers. ChatGPT and other LLMs have known limitations including producing incorrect or biased answers [1,7,8], and automation bias (ie, humans favoring suggestions from automated decision-making systems over their own judgment) is a key concern to watch for [28]. Liability will also be a key concern that will necessitate careful curation of chatbot responses [29].

The appropriateness of each deployment model likely depends on the clinical complexity and severity of the condition. Higher-risk or -complexity clinical interactions could use chatbots to generate drafts for clinician editing or approval and lower-risk situations may allow for direct patient-chatbot interaction. Alternatively, it may be useful to have chatbots classify questions into administrative versus health questions, replying directly to administrative questions and drafting responses for provider approval to health questions. The role and impact of the disclosure of origination (human vs chatbot) also needs further exploration, especially with regards to ethics, effectiveness, and implications for the patient-provider relationship.

Although our study addressed new questions with state-of-the-art technology, it has some key limitations. First, ChatGPT was not trained on medical data and could be inferior to medically trained chatbots such as Med-PaLM [14]. Second, there was no specialized prompting of ChatGPT (eg, to be empathetic), which can help responses sound more human and could potentially increase patients’ willingness to accept AI chatbot–generated responses [30]. Third, it is possible that individual style (of both the human provider and chatbot) can impact distinguishability, although the responses presented were for the most part short and impersonal. Fourth, it is possible that there were biases in the web-based survey since the participants were given the prior knowledge that 5 answers were human generated and 5 answers were chatbot generated. Fifth, this study was conducted using ChatGPT in January 2023 (based on GPT-3.5; OpenAI) [1]. Since then, more advanced underlying GPT models such as GPT-4 have been released, and further development has integrated GPT with EHRs and adapted it to medical tasks such as responding to patient portal messages [6]. Finally, this study used only 10 real-world questions with human responses from 1 provider. Further studies incorporating larger numbers of real-world questions and responses are warranted.

In addition, future research may explore how to prompt chatbots to provide an optimal patient experience [30], investigate if there are types of questions that chatbots are better at answering than others, and explore if patients feel more trusting if there is clinician review before chatbots respond. Continued studies investigating how model responses differ by patient demographics (eg, gender and race) [1,7,8] will be critical to ensure the recognition and mitigation of model biases and work toward equitable responses. Research to mitigate risks of AI chatbot–generated responses, including the potential for patient harm caused by incorrect answers; cybersecurity vulnerabilities [31]; and environmental, social, and financial risks [32] should also be further explored.

### Conclusion

Overall, our study shows that ChatGPT responses to patient questions were weakly distinguishable from provider responses. Furthermore, laypeople trusted chatbots to answer lower-risk health questions. It is important to continue studying how patients interact (objectively and emotionally) with chatbots as they become a commodity and move from administrative to more clinical roles in health care.

## Appendix

Multimedia Appendix 1 Distribution of correct responses.

## Abbreviations

AI: artificial intelligence
ChatGPT: Chat Generative Pre-trained Transformer
EHR: electronic health record
GPT: Generative Pre-trained Transformer
LLM: large language model

## Data Availability

The anonymized data generated during and/or analyzed during this study are available from the corresponding author on reasonable request.
