# Supplementary figures and images for: Putting ChatGPT’s Medical Advice to the (Turing) Test: Survey Study
Source: JMIR Med Educ. 2023 Jul 10;9:e46939. doi: 10.2196/46939 (PMC10366957; doi:10.2196/46939)

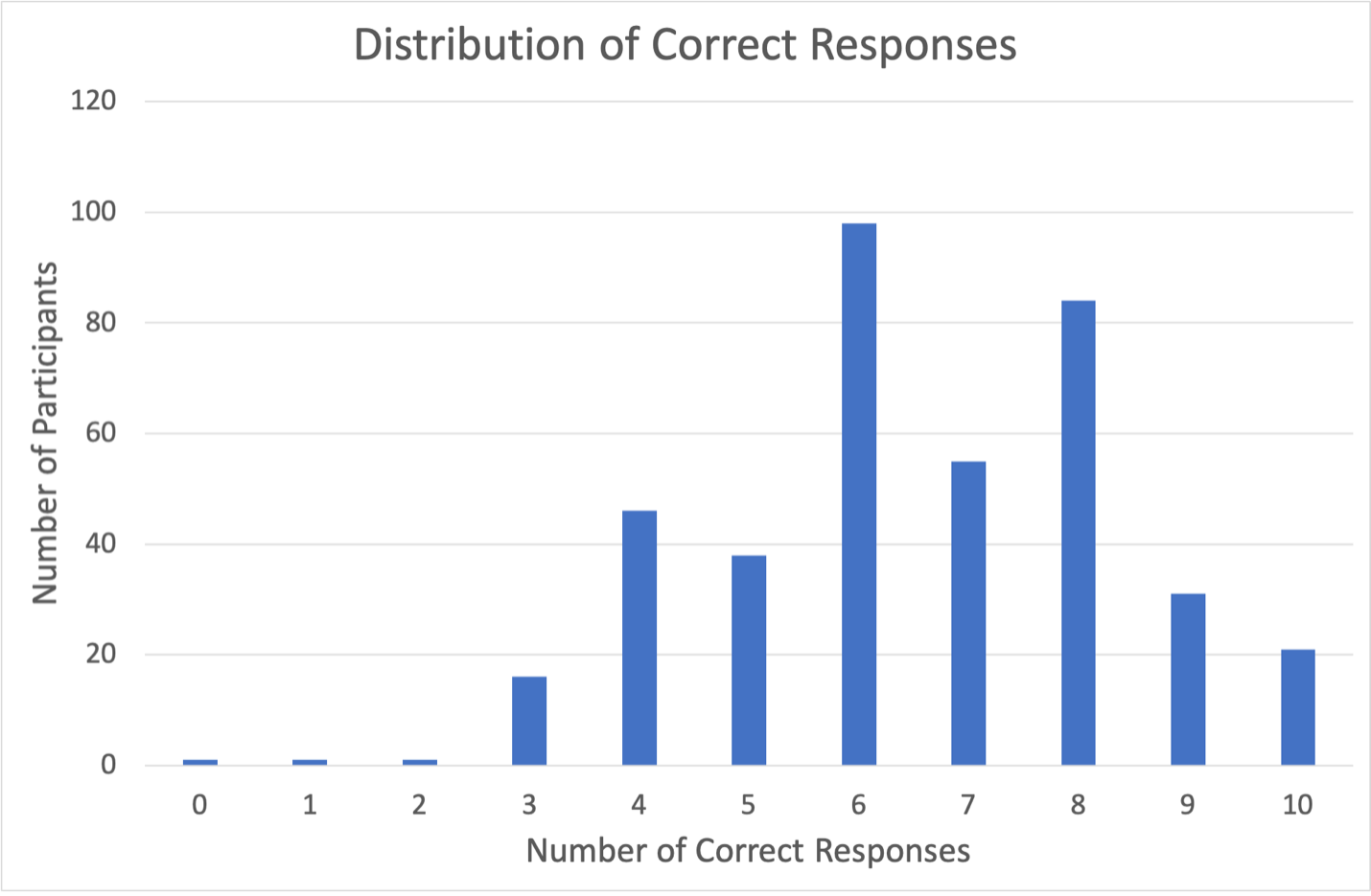

Supplement: Multimedia Appendix 1 [file mededu_v9i1e46939_app1.png]
